# Repetitive Peripheral Magnetic Nerve Stimulation (rPMS) as Adjuvant Therapy Reduces Skeletal Muscle Reflex Activity

**DOI:** 10.3389/fneur.2019.00930

**Published:** 2019-08-27

**Authors:** Volker R. Zschorlich, Martin Hillebrecht, Tammam Tanjour, Fengxue Qi, Frank Behrendt, Timo Kirschstein, Rüdiger Köhling

**Affiliations:** ^1^Faculty of Philosophy, Institute of Sports Science, University of Rostock, Rostock, Germany; ^2^Department of Ageing of Individuals and Society, Faculty of Interdisciplinary Research, University of Rostock, Rostock, Germany; ^3^Department of Sport Science, University of Oldenburg, Oldenburg, Germany; ^4^Department of Psychology and Neurosciences, Leibniz Research Centre for Working Environment and Human Factors, Technical University Dortmund, Dortmund, Germany; ^5^Department of Sport Training, Sport Coaching College, Beijing Sport University, Bejing, China; ^6^Reha Rheinfelden, Research Department, Rheinfelden, Switzerland; ^7^Oscar-Langendorff-Institute of Physiology, University Medicine Rostock, Rostock, Germany

**Keywords:** muscle spasticity, muscle hypertonia, pain, cerebral palsy, reflex, magnetic stimulation

## Abstract

**Background:** The reduction of muscle hypertonia and spasticity, as well as an increase in mobility, is an essential prerequisite for the amelioration of physiotherapeutical treatments. Repetitive peripheral magnetic nerve stimulation (rPMS) is a putative adjuvant therapy that improves the mobility of patients, but the underlying mechanism is not entirely clear.

**Methods:** Thirty-eight participants underwent either an rPMS treatment (*N* = 19) with a 5 Hz stimulation protocol in the posterior tibial nerve or sham stimulation (*N* = 19). The stimulation took place over 5 min. The study was conducted in a pre-test post-test design with matched groups. Outcome measures were taken at the baseline and after following intervention.

**Results:** The primary outcome was a significant reduction of the reflex activity of the soleus muscle, triggered by a computer-aided tendon-reflex impact. The pre-post differences of the tendon reflex response activity were −23.7% (*P* < 0.001) for the treatment group. No significant effects showed in the sham stimulation group.

**Conclusion:** Low-frequency magnetic stimulation (5 Hz rPMS) shows a substantial reduction of the tendon reflex amplitude. It seems to be an effective procedure to reduce muscular stiffness, increase mobility, and thus, makes the therapeutic effect of neuro-rehabilitation more effective. For this reason, the 5 Hz rPMS treatment might have the potential to be used as an adjuvant therapy in the rehabilitation of gait and posture control in patients suffering from limited mobility due to spasticity. The effect observed in this study should be investigated conjoined with the presented method in patients with impaired mobility due to spasticity.

## Introduction

Reducing muscle hypertonia or spasticity in order to regain independent mobility is an essential goal of a physiotherapeutical treatment in neuro-rehabilitation. The rapid normalization of the muscle tone is a criterion that can crucially influence the outcome of future rehabilitation or in training programs. An increase of hypertonia and stiffness in skeletal muscles is a common phenomenon that is associated with considerable discomfort or pain and refers to both chronic and acute cases, e.g., in post-stroke rehabilitation (1), or might also occur after strength training (2–4). Some form of intervention is, therefore, required, in case of spasticity interferes with function, or where long-term complications are expected (5). Based on the knowledge of the mechanisms responsible for muscular hypertonia, one option for patients could be a targeted treatment with repetitive peripheral magnetic nerve stimulation (rPMS), as adjuvant therapy that could improve the mobility of patients or athletes.

Some studies have been conducted to describe the implications of rPMS (reducing hypertonia, spasticity, and so forth) on spinal neuromuscular structures [for review (6)]. By employing a pulsed magnetic stimulation of the peripheral nerve a twitch can be triggered in a specific skeletal muscle. This twitch contraction is achieved initially through the triggering of an action potential in the associated motor nerve (7). The axon of the α-motor neuron conducts the generated action potential to the neuromuscular junction, thus triggering a twitch contraction in the muscle. A rPMS caused muscle activation is induced by a nerve stimulation and not by direct muscle activation (8). Depending on the stimulation frequency, the repetitive stimulation of the nerve can lead to muscle contraction in such a way that the individual twitches merge on higher stimulation frequencies. In this experiment, the magnetic stimulation was performed at the branches of the posterior tibial nerve, which resulted in an unfused twitch contraction of the triceps surae muscle. The stimulation frequency was chosen to be low enough, at five pulses per second, so that no complete merging of the individual twitches was observed.

The effect of a magnetic stimulation, especially on the peripheral nerves, has been described by some authors (9–17). Transcutaneous electrical nerve stimulation (TENS) shows some efficacy in treating spasticity (18, 19), as measured by a modified clinical Ashworth-scale. A significant reduction of the muscle tone after a TENS treatment (decrease in resistive torque) was observed (20), but it was not always associated with a decrease in reflex activity. In contrast to TENS, magnetic pulses can be applied painlessly to the efferent motor nerves at higher intensities, since the cutaneous receptors and their nerves are not stimulated to the same extent. Furthermore, rPMS can stimulate deeper nerve structures that cannot be targeted with TENS. It is of particular importance, in terms of a painless use in therapeutic rPMS applications, that the activation of the afferent sensory nerves is significantly lower with a magnetic stimulation (21, 22). A painless treating of motor nerve structures with a magnetic stimulation is possible. Whereas, the treating of peripheral nerves with an electric stimulation gives rise to significant pain symptoms (23–25). Different rPMS protocols have been used to successfully treat skeletal muscle spasticity, which could be defined by an increase of phasic and tonic stretch reflex activity, depending on the velocity of the muscle stretch (26). In an earlier study, it was found that it was possible to improve electrophysiological measures of spasticity by using a biphasic 12 Hz rPMS protocol for 8 s, followed by a 22 s rest, for a total of 30 min stimulation (27). Stimulations with 15 Hz of 3 s duration and 30 trains, with an inter-train-interval of 2 s, with 1,350 impulses in total, and with an intensity of 60 A/μs (40% of maximal stimulator intensity), applied to the rectus femoris muscle (28), or the triceps surae muscle complex (29), results in different effects.

A magnetic stimulation, with a 20 Hz repetition rate, was used by Struppler et al. (30) and Marz-Loose and Siemes (31), but with a different amount of impulses (5,000 and 2,000, respectively), and with different stimulation sites. A 50 Hz rPMS protocol was used to decrease spasticity (32) in six sessions, with a continuous theta-burst of 200 ms, at an inter-stimulus interval of 5 sec^−1^, with a repetition rate in a 60 s train, with 900 impulses and an intermittent theta-burst. The theta-mode consisted of 2 s trains, repeated every 10 s, with 900 impulses (300 s). The described intermittent theta-burst stimulation produced a cyclic activation-relaxation of the muscle (33).

A low-frequency stimulation with 3 Hz (34) was applied as an accompanying curative treatment to physiotherapy in post-stroke rehabilitation. This low- frequency rPMS was performed with 600 stimuli, in a series of 3 s, followed by 3 s rest, at a 60% intensity. All studies except one (29) reported positive clinical or physiological effects of the rPMS treatment. Despite these findings, the mechanisms of treating muscle tone, clonus, and spasticity, are still poorly understood (35, 36) and they are not ideally measurable, either mechanically (37), or by using clinical scales (38). A study of the reflex responses provides a quantitative representation of the effects of an rPMS treatment. One of the central questions in this context is: “under what conditions can the spinal motor circuits be influenced by means of targeted magnetic pulses?” Thus, the purpose of this study was to investigate the effect of 5 Hz rPMS on the regulatory spinal circuits. The underlying hypothesis was the following: the researchers presumed that the application of repetitive low-frequency pulsed magnetic fields to the peripheral nerves of the muscle would reduce the compound muscle action potential (CMAP) amplitudes of the tendon-reflex (T-reflex) activities of the soleus muscle. Since the measurement of muscle stiffness or spasticity, biomechanically encounters great difficulties, an investigation of the peripheral reflex processes serves as a quantifiable marker (31). A validation of the study's hypothesis, as given in the present paper, opens up the possibility of introducing rPMS, in order to treat the phenomenon of muscle hypertonia, in a wide range of applications in physiotherapy, and in the various forms of muscle training. To the best of the authors' knowledge, this study is the first, which addresses the effects of rPMS on tendon reflex response behavior of the soleus muscle in quantitative detail, and it provides a quantifiable parameter of muscle relaxation.

## Methods

### Ethics Statement

The study was approved by the local ethics committee of the medical faculty of the University Rostock, Germany (Identifier No. A20160052), as required by the international standards of the *Declaration of Helsinki* (39). All participants gave written informed consent prior participation.

### Participants

The study involved 38 subjects (40). Of these, 19 volunteers participated in the treatment group (TG; 12 men/7 women), with a mean age of 25.4 (±3.0), a mean height of 177.3 cm (±8.9 cm), and a mean body weight of 73.3 kg (±13.5 kg). This was while 19 subjects (14 men/5 women) participated in the control group (CG; sham stimulation), with a mean age of 27.5 (±4.4), a mean height of 177.1 cm (±8.6 cm), and a mean body weight of 72.3 kg (±10.0 kg). Those cases with metallic implants were excluded from the study (41). All of the subjects were healthy students and staff members, with unremarkable lower extremities, both in orthopedic and neurological terms, from whom informed consent was obtained (42). The participants had ample opportunities to familiarize themselves with the experiment and with the treatment.

### Study Design

The investigation was conducted in a pre-test post-test design with matched groups. To rule out every other influence on the study and a reflex modulation beside the rPMS treatment, for example, a response decrease in the course of the experiment (43), we chose a control group design. In the control group, the identical procedure was performed as in the treatment group except the stimulator did not generate a magnetic field. The experiment initially involved the measurement of the Achilles tendon-reflex responses of the soleus muscle, by means of a linear-actuator reflex hammer (fifteen measurement trials) in a sitting position. The subjects were asked to maintain a right angle, both at the hip and at the knee joint during the reflex measurements. The tendon taps were applied with a time interval of at least 10 s in between, followed by 5 Hz rPMS, based on the protocol as described below. The stimulation was applied in a standing position to the posterior tibial nerve in the popliteal fossa, resulting in contractions of the triceps surae muscle. The magnetic stimulation of peripheral nerves is somewhat unfocal. We have tried to exclude any cocontraction with palpation of the tibial anterior muscle simultaneously. The subjects maintained a slight tension in the stimulated muscle over the entire stimulation period. In the third part of the experiment, the subjects were re-investigated in the sitting position, in order to ensure an identical reflex triggering. The re-examination of the T-reflex responses of the soleus muscle was carried out as described. Care was taken for the exact repositioning of the participants for the post measurements. Throughout the entire reflex measurements, complete relaxation of the soleus muscle was carefully controlled via online electromyography (EMG). Trials with a muscular activity >50 μV in a period of 100 ms prior to the reflex triggering were discarded. In order to check the uniformity of the experimental conditions when triggering the reflex responses, the impact forces were tested likewise. All of the impact forces were evaluated according to the peak values of the reflex hammer's impact.

### Tendon Reflex

The T-reflex excitability of the soleus muscle was probed by a brisk mechanical impact elicited by a linear-actuator (Copley Controls, Canton, MA 02021, USA) at the Achilles tendon, with a thrust rod being moved in the acceleration mode. The actuator showed perfect repeatability and produced an absolute position accuracy of 0.35 mm. The control parameters were chosen as follows: (i) the reflex hammer (head of the thrust rod) exactly covered a distance of 40 mm from the home position to the impact point on the Achilles tendon; (ii) the home position of the actuator was defined by a contact of the reflex hammer at the Achilles tendon; and (iii) the development of the maximum impact force occurred within <12 ms, triggering supra-maximal reflexes (44). A tendon vibration due to the impact, as described (45), could not be detected when using the brisk actuator impacts.

**Figure 1:**
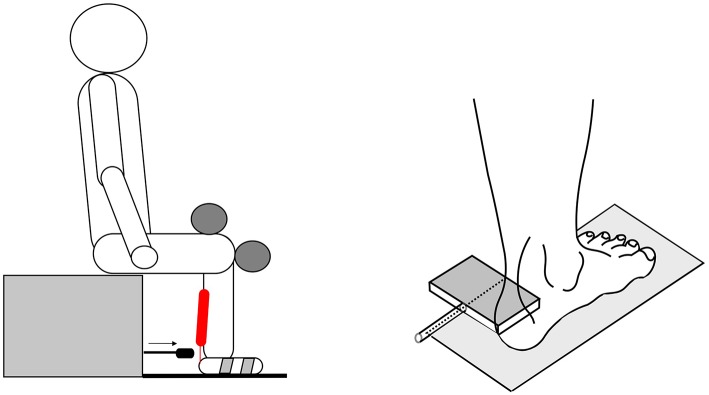
The sketch shows the subject had a fixed sitting position. The subjects were seated comfortably upright, with a knee angle and a foot angle each of 90°. The left foot was fixed with two stirrups on a tempered (30°C) footplate. The upper leg was secured at the topside; the arm and head positions were kept constant during the whole experiment. The reflex-triggering hammer (right side) was equipped with a force sensor, in order to record the impact forces at the muscle-Achilles tendon-complex.

The T-reflex of the soleus muscle was triggered in the sitting position, via an impact at the Achilles tendon (Figure 1). The level of the impact force depended individually on the elasticity of the biological structures (tendon, muscle, subcutaneous fatty tissue), with a minimum of 40 N, up to a maximum of 100 N, between the subjects. While there was some inter-individual variability, it was possible to trigger fairly uniform impacts in any given subject. The machine-controlled tendon taps produced constant supra-maximal impacts and elicited constant reflex responses. Methodologically, there was an important advantage in investigating the muscular relaxation via the T-reflex activity, as a measure at an interval scale level, in comparison to the commonly used Ashworth Scale (38).

### Force Measurement

The force was measured during the impact that triggered the entire measuring process. The triggering threshold was 7 N, as accurately defined by the home position of the linear actuator. The reflex hammer fixed at the head of the thrust rod of the programmable linear actuator was equipped with a piezoelectric force sensor (Type 9011A, Kistler Instruments, Winterthur, Switzerland) and it measured the impact force at the Achilles tendon. The force-sensor data was prepared for the online presentation by using a Kistler charge amplifier type 5037A. The constant and comparable triggering of the reflex responses required an exact repositioning of the impact point at the tendon.

### Electromyography—Recording and Sampling

The reflex responses were recorded by using a bipolar montage (46) of the cup-electrodes (HELLIGE baby-electrodes; GE medical systems, Milwaukee, USA), with an electrode area of about 12 mm^2^ (Ag/AgCl) and an inter-electrode distance of 20 mm, placed longitudinally over the belly of the lateral soleus muscle. The skin preparation procedures were applied before the electrode application, i.e., cleaning the skin with alcohol and hair-removal before the positioning of the electrodes. An electrode gel (HELLIGE, GE medical systems, Milwaukee, USA) was used to ensure optimal skin-electrode contact. The electrodes and the twisted wires were fixed to the skin with adhesive tape. The surface electromyograms were recorded with a custom-made differential-amplifier (x1000 amplification, input resistance 16 GΩ). The signals were recorded with a DAQ-Card 6024 (National Instruments, Austin, Texas, USA) at a 12-bit resolution and at a sampling rate of 10,000/s, using the DIAdem 8.1 (National Instruments, Ireland) signal processing program. The movement artifacts in the EMG were filtered with a Butterworth 2nd-order high-pass filter with a cut-off frequency of 5 Hz (47).

### Magnetic Stimulation

The pulsed magnetic stimulation was carried out with a Magpro 30+ stimulator with the Mag-Option (MagVenture, Skovlunde, Denmark—formerly Medtronic) and a parabolic coil type MMC-140, with the convex side being used. The stimulator generated biphasic symmetric pulses, with a duration of 280 μs, and a magnetic flux density of a maximal 4.5 Tesla. The stimulation protocol that was chosen from preliminary tests was performed with a stimulus intensity of 60% of the maximum stimulator output, corresponding to a current flux of 94 A/μs. The stimulation intensity was the same for each subject and was clearly visible above the motor threshold. The stimulation was carried out with bursts of 15 stimuli each, at 5 stimulations per second, and with 750 pulses in total. The interval between the trains was 3 s with 50 trains, and the stimulation lasted 5 min. The same procedure (coil positioning, stimulation, and the like) was conducted in the sham group, but without exposure to pulsed magnetic fields.

### Data Analysis

The peak-to-peak compound muscle action potential (CMAP_pp_) amplitude of the lateral site of the soleus muscle was recorded to characterize the reflex activity. The changes in CMAP_pp_ were measured at the baseline condition and immediately after the magnetic stimulation or the sham stimulation. The CMAP_pp_ amplitude measurements were made from the stationary EMG data (Butterworth high-pass filter). The algorithm added the absolute amplitude values from the lowest negative peak and the highest positive peak. In order to check the uniformity of the experimental conditions when triggering the reflex responses, the impact forces were observed online during each trial.

### Statistics

The effects of the 5 Hz rPMS on the measurements of the spinal tendon reflex excitability were compared in the treatment group and the control group, using a mixed design analysis of variance (ANOVA) with between the group effects and the repeated-measure effects of time as the main factor. The data was analyzed by SPSS version 20.0 for Windows (SPSS Inc., Chicago, IL, USA). All of the values in the text, and figures, are expressed as mean ± SD. A *P* < 0.05 was considered significant.

## Results

**Figure 2:**
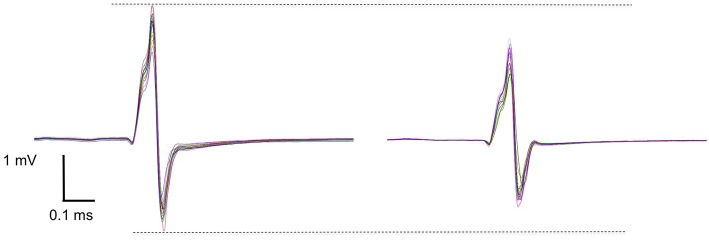
A raw data set of one subject shows the effect of before (**left**) and after (**right**) the rPMS. Each graph shows 15 peak-to-peak superimposed compound muscle action potential (CMAP_pp_) curves that were induced by a reflex hammer impact on the Achilles tendon. Mean CMAP_pp_ amplitudes were ~4.2 mV at baseline and ~3.5 mV after the rPMS in this subject.

The reflex response behavior of the skeletal musculature after the rPMS was measured in terms of the tendon tap triggered CMAP_pp_ amplitude values. The CMAP_pp_ amplitudes decreased after 5 Hz rPMS stimulation in the treatment group (Figure 2).

**Figure 3:**
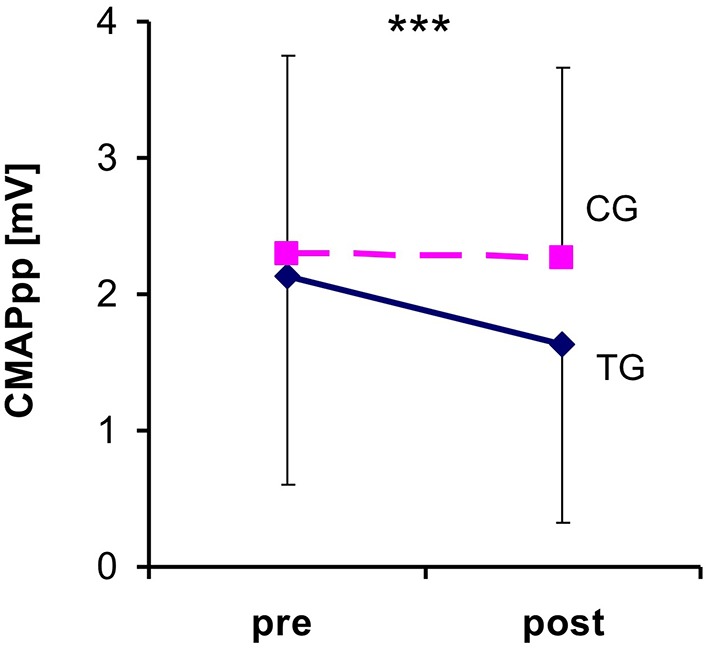
Changes in the compound muscle action potential (CMAP_pp_) size before the repetitive peripheral magnetic stimulation (rPMS) and post stimulation. The dashed line represents the control group (CG) and the continuous line indicates the treatment group (CG). Each bar corresponds to the SD value. Note that the reflex responses significantly decreased in size only after the rPMS in the treatment group. ***Denotes a significant difference between pre- and post-test (***P ≤ 0.001) in CG.

The ANOVA for repeated measures showed a significant interaction between the time and group [*F*_(1, 36)_ = 16.789; *P* ≤ 0.001; η_p_^2^ = 0.318]. The *post-hoc* analysis with a Bonferroni adjustment demonstrated that the CMAP_pp_ amplitude significantly decreased after the 5 Hz rPMS treatment when compared to the baseline measurements (*P* ≤ 0.001). The CMAP_pp_ amplitudes of the treatment group were 2.13 ± 1.54 mV in the pre-test and 1.63 ± 1.30 mV in the post-test, which meant a reduction of 23.7% (Figure 3). In contrast, the CMAP_pp_ amplitudes of the control group revealed no significant differences (*P* ≤ 0.390) from the pre-test (2.30 ± 1.45 mV) to the post-test (2.26 ± 1.40 mV) and the change rates were−1.74%.

## Discussion

The present paper demonstrates that a repetitive magnetic nerve stimulation (rPMS) with 750 stimuli at a stimulation frequency of 5 Hz reduces the T-reflex response of the soleus muscle. After the rPMS treatment, a substantial and significant reduction in the normalized CMAP_pp_ amplitude after triggering the Achilles tendon reflex by −23.7% was found, in contrast to the control group (−1.74%). The pulsed magnetic stimulation of the muscles acted on the control state of the neuromuscular circuits; the reduction in the CMAP amplitude was apparently attributed to a significantly decreased excitation of the T-reflex triggered α-motor neurons. This means that the rPMS treatment of a muscle reduces a significant number of α-motor neurons from the triggering of an action potential. Presynaptic and/or postsynaptic processes may have influenced this.

The reason for the occurrence of such reflex inhibition after the treatment must remain unclear at this time since the functional processes of rPMS have not yet been fully explored. It is not known, for example, whether the effects of the magnetic stimulations take place via an orthodromic or an antidromic stimulation of the peripheral nerves. In the first case (orthodromic), the stimulation could directly act on the axons of the α-motor neurons and in the second case (antidromic), with a partial inactivation of the axon hillock. Alternatively, the stimulation could act on the axons of statically or dynamically oriented γ-motor neurons, or the muscle spindles, which would then induce secondary and indirect effects via spindle sensitivity changes. In the latter case (antidromic motor nerve stimulation), a reflex change via activation of a Renshaw inhibition is conceivable but not likely (48). Some investigations are hinting and speaking in favor of an effect being exerted on the γ-motor regulatory circuits by the magnetic stimulation, also influencing the tonus part of the γ-motor sensory system. This could, in part, explain the researchers' previous results (29), where no critical reduction of H-reflex activity was found after the rPMS, in accordance with the findings of Goulet et al. (19), who likewise found no substantial reduction of H-reflex activity after a TENS.

**Figure 4:**
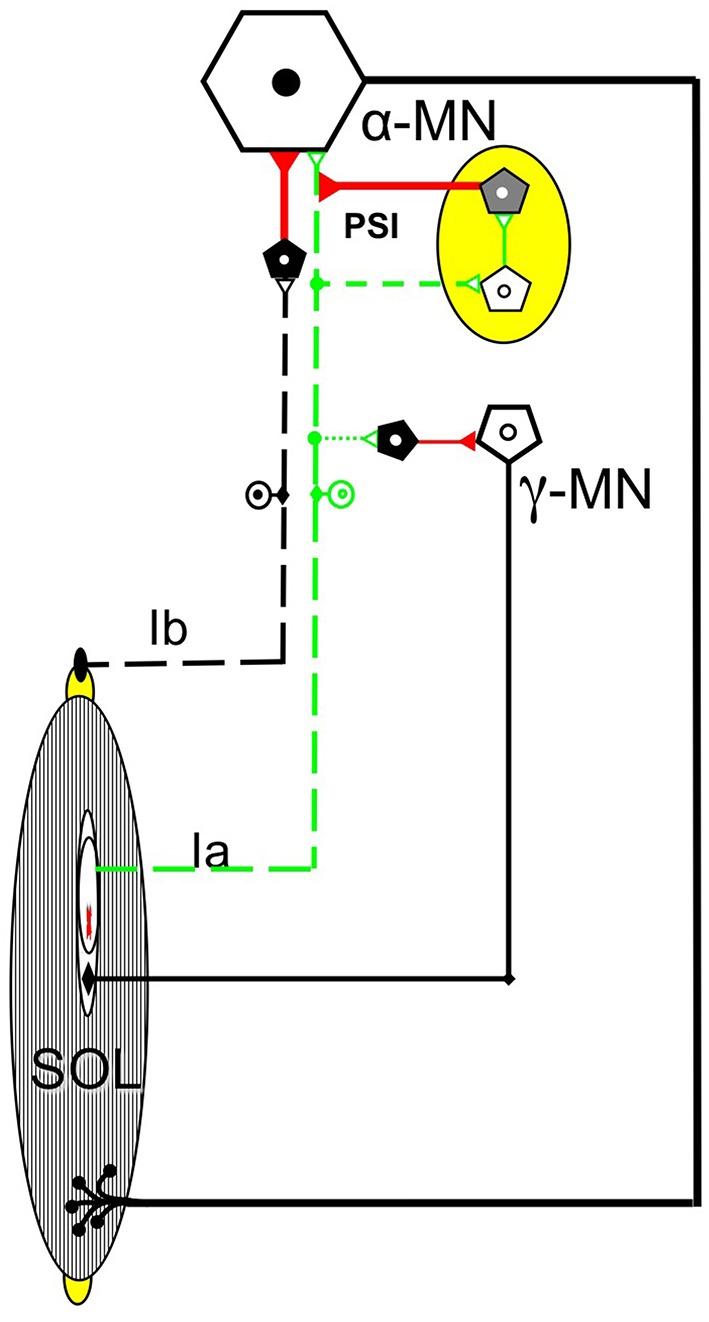
The diagram shows the spinal circuitry that was possible involved γ-motor neurons (γ-MN), α-motor neurons (α-MN), Ia afferents (Ia), Ib afferents (Ib), or presynaptic mechanism (PSI), by near-muscle magnetic nerve stimulation. However, at this time, no conclusions can be reached about the mode of action of the magnetic stimulation on the spinal neuronal structures.

Thus, the sensory system's sensitivity appeared to decrease markedly. It is reasonable that this may be the explanation for the efficacy of an rPMS treatment. It is likely that the influence exerted by the magnetic stimulation of the tibial nerve caused a sensitivity reduction of the γ-motor regulatory circuit (see Figure 4). However, a conclusive differentiation among the possibilities as mentioned above would necessitate pharmacological interventions, which are impossible to obtain in healthy human subjects.

These findings might have a specific implication for clinical use, as there is varying evidence for the existing physiotherapeutic and pharmacological treatment approaches in the therapy of spastic syndrome. It is described in the literature, for instance, that there is a basically positive effect of a physiotherapeutical intervention (49), which is on the one hand, superior to a drug treatment alone, because of the side effects (50), but on the other hand, this still needs to be further investigated. Furthermore, anti-spastic drugs can improve spasticity on corresponding scales (e.g., on a modified Ashworth Scale), but the treatment is often not associated with an improvement in everyday activities. There was also no significant improvement in the gait parameters, as a result of the Botulinum toxin type A injection into the calf musculature (51, 52). In a combination with physiotherapy (53) or TENS (54, 55), positive effects were reported, which in turn, is still not sufficient for an unequivocal recommendation. However, in addition to controlled studies with a large number of subjects, systematic investigations of the dose-effect relationships of the various intervention combinations are lacking. Against this background, there is still room for new therapeutical methods. Based on this study's results, rPMS could indeed have a promising effect as an adjunct to other therapeutical methods. The duration of the outcome in healthy subjects and patients, the impact in a combination with other treatment approaches, and the usability and the feasibility in a clinical setting, should all be evaluated conjoined for a potential translation of rPMS into clinical practice with a quantitative tendon reflex measurement.

This study has some limitations. The stimulation of a peripheral nerve structure is not very focal. We used a large magnetic coil over the popliteal fossa, which limits specificity of stimulation in the posterior tibial nerve. It can not be ruled out that this peripheral stimulation causes further nerve structures to be excited. For this reason, we took care of the exact stimulation response and the fact that antagonistic muscles do not co-contract during the stimulation also. However, we cannot exclude subliminal influences on surrounding nerve structures (56). We only investigated healthy subjects, so it would be valuable to further verify whether the T-reflex is also altered in patient populations and whether that change is consistent with the positive effects found in the clinical assessments performed in earlier studies (34).

## Conclusion

The results have demonstrated that the T-reflex was reduced after 5 Hz rPMS. This relaxing effect on the musculature was investigated in this experiment indirectly, by using the Achilles tendon reflex to represent the decrease in excitability of the α-motor neurons or the presynaptic effects. The artificial relaxation of the skeletal musculature has excellent practical benefits in a wide variety of fields. The use of rPMS in physiotherapy and neuro-rehabilitation is an important area where it can have significant effects; the therapeutical advances in spasticity and muscle hyperreflexia may also be achievable. In addition, the skeletal musculature tone can be reduced significantly after muscle training through an rPMS procedure.

## Data Availability

The datasets analyzed in this manuscript are not publicly available. Requests to access the datasets should be directed to volker.zschorlich@uni-rostock.de.

## Author Contributions

The experiments were conducted in the Laboratory of the Department of Movement Science, Institute of Sport Science, University of Rostock, Germany. VZ and MH contributed to the conception and the design of the experiment. FQ, TT, and VZ collected the data. VZ, FB, FQ, and MH contributed to the analysis and the interpretation of the data. VZ, FB, FQ, MH, TK, and RK drafted the paper and revised it critically for important intellectual content.

### Conflict of Interest Statement

The authors declare that the research was conducted in the absence of any commercial or financial relationships that could be construed as a potential conflict of interest.

## Abbreviations

ANOVA: analysis of variance
CMAP_pp_: compound muscle action potential (peak-to-peak)
EMG: Electromyography
rPMS: repetitive peripheral magnetic stimulation
TENS: transcutaneous electrical stimulation
(T-reflex): tendon reflex.
